# Clinical Outcome Prediction in COVID-19 Patients by Lymphocyte Subsets Analysis and Monocytes’ iTNF-α Expression

**DOI:** 10.3390/biology10080735

**Published:** 2021-08-01

**Authors:** Gabriele Madonna, Silvia Sale, Mariaelena Capone, Chiara De Falco, Valentina Santocchio, Tiziana Di Matola, Giuseppe Fiorentino, Caterina Pirozzi, Anna D’Antonio, Rocco Sabatino, Lidia Atripaldi, Umberto Atripaldi, Marcello Raffone, Marcello Curvietto, Antonio Maria Grimaldi, Vito Vanella, Lucia Festino, Luigi Scarpato, Marco Palla, Michela Spatarella, Francesco Perna, Pellegrino Cerino, Gerardo Botti, Roberto Parrella, Vincenzo Montesarchio, Paolo Antonio Ascierto, Luigi Atripaldi

**Affiliations:** 1 Melanoma, Cancer Immunotherapy and Development Therapeutics Unit, Istituto Nazionale Tumori IRCCS Fondazione G. Pascale, 80131 Napoli, Italy; g.madonna@istitutotumori.na.it (G.M.); me.capone@istitutotumori.na.it (M.C.); m.curvietto@istitutotumori.na.it (M.C.); a.grimaldi@istitutotumori.na.it (A.M.G.); v.vanella@istitutotumori.na.it (V.V.); l.festino@istitutotumori.na.it (L.F.); l.scarpato@istitutotumori.na.it (L.S.); m.palla@istitutotumori.na.it (M.P.); 2 UOC Biochimica Clinica, AORN Ospedali dei Colli—Monaldi—Cotugno—CTO, 80131 Napoli, Italy; silviasale@libero.it (S.S.); chia.defalco@gmail.com (C.D.F.); valentina83.sa@libero.it (V.S.); dimatola@unina.it (T.D.M.); caterinapirozz@virgilio.it (C.P.); anna.dant@libero.it (A.D.); rocco_sabatino@virgilio.it (R.S.); luigi.atripaldi@ospedalideicolli.it (L.A.); 3 UOC Fisiopatologia e Riabilitazione Respiratoria, AORN Ospedali dei Colli—Monaldi—Cotugno—CTO, 80131 Napoli, Italy; giuseppe.fiorentino@ospedalideicolli.it; 4 Dipartimento di Scienze Mediche Traslazionali, University of Campania “Luigi Vanvitelli”, 80138 Naples, Italy; lidia.atripaldi@studenti.unicampania.it (L.A.); umberto.atripaldi@studenti.unicampania.it (U.A.); 5 UOC Microbiologia e Virologia, AORN Ospedali dei Colli—Monaldi—Cotugno—CTO, 80131 Napoli, Italy; marcello.raffone@ospedalideicolli.it; 6 UOSD di Farmacia, AORN Ospedali dei Colli—Monaldi—Cotugno—CTO, 80131 Napoli, Italy; michela.spatarella@ospedalideicolli.it; 7 Dipartimento di Medicina Clinica e Chirurgia, Università degli Studi di Napoli “Federico II”, 80131 Naples, Italy; francesco.perna@ospedalideicolli.it; 8 Istituto Zooprofilattico Sperimentale del Mezzogiorno, 80055 Portici, Italy; stategia@izsmportici.it; 9 Scientific Direction, Istituto Nazionale Tumori IRCCS Fondazione G. Pascale, 80131 Napoli, Italy; g.botti@istitutotumori.na.it; 10 UOC Malattie Infettive ad Indirizzo Respiratorio, AORN Ospedali dei Colli—Monaldi—Cotugno—CTO, 80131 Napoli, Italy; roberto.parrella@ospedalideicolli.it; 11 UOC Oncologia, AORN Ospedali dei Colli—Monaldi—Cotugno—CTO, 80131 Napoli, Italy; vincenzo.montesarchio@ospedalideicolli.it

**Keywords:** COVID-19, iTNF-α, SARS-CoV-2, monocytes, neutrophils, eosinophils, lymphocytes, tregs

## Abstract

**Simple Summary:**

Several studies have explored the role of the inflammatory cells and cytokines involved in the protection or pathogenesis of coronavirus disease 2019. Unfortunately, the results have been controversial, and further studies are needed to better understand not only the roles but also the balance of these parameters, which are crucial data to improve prevention and treatment. As COVID-19 has a well-determined phasic progression and rapidly deteriorates approximately seven days after the onset of symptoms, it is extremely necessary to detect the clinical signs that are predictive of the outcome as early as possible. To this end, in this preliminary study, we evaluated the data relating to the monocyte intracellular TNF-α expression and lymphocyte subpopulations in peripheral blood collected from patients at admission and every day of hospitalization until day 7. Our findings point to a modulation of the different cellular mediators of the immune system, which probably play a key role in the outcome of the coronavirus disease 2019.

**Abstract:**

In December 2019, a novel coronavirus, “SARS-CoV-2”, was recognized as the cause of coronavirus disease 2019 (COVID-19). Several studies have explored the changes and the role of inflammatory cells and cytokines in the immunopathogenesis of the disease, but until today, the results have been controversial. Based on these premises, we conducted a retrospective assessment of monocyte intracellular TNF-α expression (iTNF-α) and on the frequencies of lymphocyte sub-populations in twenty-five patients with moderate/severe COVID-19. We found lymphopenia in all COVID-19 infected subjects compared to healthy subjects. On initial observation, in patients with favorable outcomes, we detected a high absolute eosinophil count and a high CD4+/CD8+ T lymphocytes ratio, while in the Exitus Group, we observed high neutrophil and CD8+ T lymphocyte counts. During infection, in patients with favorable outcomes, we observed a rise in the lymphocyte count, in the monocyte and in Treg lymphocyte counts, and in the CD4+ and in CD8+ T lymphocytes count but a reduction in the CD4+/CD8+ T lymphocyte ratio. Instead, in the Exitus Group, we observed a reduction in the Treg lymphocyte counts and a decrease in iTNF-α expression. Our preliminary findings point to a modulation of the different cellular mediators of the immune system, which probably play a key role in the outcomes of COVID-19.

## 1. Introduction

In late December 2019 in Wuhan, Hubei Province, China, several patients with severe pneumonia of unknown origin were reported from different hospitals [1]. Immediately, the Chinese Center for Disease Control and Prevention dispatched an expert team to assist Hubei health authorities and to conduct epidemiological and etiological investigations [1]. The following month, a novel coronavirus provisionally named the 2019 novel coronavirus (2019-nCoV) was identified in samples of bronchoalveolar lavage fluid (BALF) and was sequenced with use of a combination of next-generation sequencing methods (Sanger, Illumina and Nanopore) [2]. The novel coronavirus was recognized as the cause of COVID-19 disease (coronavirus disease 2019) and, considering the 88% likeness to two bat-derived severe acute respiratory syndrome (SARS)-like coronaviruses, it was defined as “SARS-CoV-2” (Severe Acute Respiratory Syndrome-Coronavirus 2) [3]. It is a systemic viral infection with a significant impact on the hematopoietic system and hemostasis as well as on the immune system. The clinical spectrum of COVID-19 ranges from asymptomatic infection to critical illness and results in high rates of hospitalization and intensive care unit (ICU) admission. The most common symptoms observed in infected patients are fever (98%), cough (76%), dyspnea (55%), myalgia, or fatigue (44%), and less common symptoms are sputum production (28%), headache (8%), hemoptysis (5%), and diarrhea (3%) [4]. In addition, as of late, there have been several reports of cases of COVID-19 presenting with the alteration of the sense of smell and taste across the world, and in particular, a European study of about 400 COVID-19 patients reported olfactory and gustatory dysfunctions in about 85% and 88% of patients, respectively [5]. Data from The Chinese Center for Disease Control and Prevention has shown that the majority of infected patients develop mild symptoms (81%; i.e., mild or nonpneumonia); 14% develop severe symptoms, i.e., dyspnea, respiratory rate ≥ 30/min, blood oxygen saturation ≤ 93; and 5% develop critical symptoms, i.e., respiratory failure, septic shock, and/or multiple organ dysfunction or failure [6]. The severity of the infection can (seen in 50% of patients) induce an excessive inflammatory reaction called cytokine release syndrome (CRS), which is also known as macrophage activation syndrome (MAS) or secondary haemophagocytic lymphohistiocytosis (sHLH) [7,8,9,10]. It represents a hypercytokinemia, and in subjects with comorbidities, it triggers severe lung inflammation that can lead to respiratory distress, sometimes with a fatal outcome [11]. Several studies have described the biological and immunological characteristics of previous coronavirus epidemics [12,13], and recent flow cytometry-based studies have shown that morphological and inflammation-related immunophenotypic changes in peripheral blood monocytes may correlate to COVID-19 severity and clinical outcome [14]. The coronavirus family, including SARS-CoV-2, induces an impairment of the cellular immune response during SARS-CoV-2 infection, with low macrophages and CD4 cytopenia functionality, defective NK cell functions, and T-cell exhaustion as well as inappropriate type I IFN responses and massive inflammatory cytokine production [15,16,17]. SARS-CoV-2 seems to induce an initial silencing of innate immunity by the suppression of INF Type 1, and subsequently, a second cytokine storm under the control of INF-γ, would induce the production of TNF-α, IL-1α, IL-1β, IL-2, IL-6, IL-7, IL-10, G-CSF, MPC1, MPI1A in the macrophages [18,19]. In particular, TNF-α is a cytokine that is closely associated with the regulation of innate immunity, inflammation, apoptosis, the inhibition of tumorigenesis, and viral replication [20]. Several studies have evaluated the potential of anti-TNF therapies with in-vivo sepsis models, highlighting how early administration can increase survival [21,22,23,24]. However, even if the TNF-α activation of endothelial cells and leukocytes and the downstream signaling pathways may contribute to an inflammatory insult, they are still fundamental for the defense of the host. In fact, TNF-α knockout models have shown a crucial role of TNF-α in host defense in viral infections, and TNF-α inhibition is associated with poor prognosis and death in different animal models [25,26,27]. Based on these premises, we conducted an initial assessment of intracellular TNF-α (iTNF-α) expression on activated monocytes and, as described in the literature, differences in lymphocyte subpopulations and laboratory indicators, which are important parameters in the delineation of the severity of the infection [28,29,30,31,32,33]. Furthermore, as recent preliminary data following the COVID-19 outbreak have indicated an association with complete blood count (CBC) parameters [34], we also explored the differences in absolute lymphocyte count (ALC), absolute neutrophil count (ANC), absolute monocyte count (AMC), absolute eosinophils count (AEC), platelets (PLT), the neutrophil-to-lymphocyte ratio (NLR) platelet-to-lymphocyte ratio (PLR), and in the frequencies of T, B, NK, and Treg lymphocyte subpopulations in patients with moderate/severe COVID-19.

## 2. Materials and Methods

### 2.1. Study Population and Sample Collection

A total of twenty-five patients hospitalized between 4 March 2020 and 12 April 2020 for respiratory insufficiency at the ‘Azienda Ospedaliera dei Colli Monaldi-Cotugno Hospital’, Italy, diagnosed with SARS-COV-2 infection and who were negative for common respiratory pathogens were considered for this study. All patients were hospitalized according to their respiratory insufficiency and without a previous COVID-19 test. Upon admission to the hospital, they were tested for COVID-19, and they their test results showed that they were positive for COVID-19 infection (day onset corresponds to day 0 of hospitalization). As indicated by WHO guidelines, confirmation of SARS-CoV-2 infection was obtained through RT-PCR positivity via an oropharyngeal swab. Following manufacturer’s instructions on a MicrobScan real-time PCR (Nurex S.r.l., Sassari, Italy), we used the Allplex™ SARS-CoV-2 multiplex real-time PCR assay (Seegene Inc., Seoul, Korea) to detect 4 target genes: RdRP, S, and N genes specific for SARS-CoV-2 and the E gene, which is expressed in all Sarbecovirus’, including SARS-CoV-2. A positive result (i.e., a Ct less than 40) for all viral targets indicated the presence of SARS-CoV-2 RNA in the sample. Data including demographic, medical history, symptoms, signs, and laboratory findings including blood routine, lymphocyte subsets, and infection-related biomarkers were collected from patients’ medical records. The patients were classified on the basis of their outcome: the Exitus Group, which was composed of patients who never recovered from COVID-19 and who died during infection, and the Good Prognosis Group, which was composed of patients who recovered from COVID-19. The intracellular TNF-α and lymphocyte subpopulations were evaluated in peripheral blood collected from patients on alternate days. Whole blood samples were collected at admission (none of the patients received any treatment before blood sampling) and every day of hospitalization until day 7 (from day 0 to day 7, for a total of eight determinations) in tubes containing EDTA or sodium-heparin, which were then immediately analyzed using flow cytometry. Blood counts were quantified as per institutional protocols using a Sysmex XN-3000 instrument (Sysmex Europe GmbH, Hamburg, Germany). The study was conducted according to the guidelines of the Declaration of Helsinki and was approved on 8 July 2020 by the Ethical Committee of the ‘AORN Ospedali dei Colli—Monaldi—Cotugno—CTO, Napoli, Italy’, approval number AOC-0020053-2020. Informed consent was obtained from all of enrolled patients for the use of their biological samples and clinical data for the purposes of clinical research and the study of diseases.

### 2.2. Flow Cytometry Analysis

Data were acquired using a Navios 10C\3L Flow Cytometry Instrument (Beckman Coulter Inc., Brea, CA, USA) and data analysis was performed using Kaluza software (Beckman Coulter Inc., Brea, CA, USA) following the manufacturer’s instructions. Analyses of the T, B, NK, and Treg lymphocyte subpopulations were performed on whole peripheral blood collected in an EDTA vacutainer while analysis of monocytes, TNF-α, and HLA-DR expression were performed on peripheral blood samples collected in sodium-heparin. To evaluate the percentage and the absolute value of the lymphocyte subpopulations, 2 kits were used, both from Beckman Coulter Inc. (Brea, CA, USA), containing mixes of monoclonal antibodies bound to different fluorochromes and were following the manufacturer’s instructions: the AQUIOS Tetra-1 Panel kit, consisting of CD45-FITC/CD4-RD1/CD8-ECD/CD3-PC5, and the AQUIOS Tetra-2+ Panel kit, consisting of CD45-FITC/(CD56+CD16)-RD1/CD19-ECD/CD3-PC5. The subpopulation of Treg lymphocytes (CD3+ CD4+ CD25+ FoxP3+) was evaluated with the DuraClone IM Treg Tube Kit (Beckman Coulter Inc., Brea, CA, USA) following the manufacturer’s instructions. For the evaluation of the monocytes, for the percentages of iTNF-α positive and HLA-DR positive monocytes (% positive cells) among the total monocyte population, the Duraclone IF Monocyte Activation Tube Kit (Beckman Coulter Inc., Brea, CA, USA) was used, following the manufacturer’s instructions and according to the methods previously described by Monneret G. et al. [35]. The protocol provides a preliminary in vitro stimulation of the monocytes with the DurActive3 Kit, which contains LPS (Lipopolysaccharide), which activates the cells and Brefeldin A to prevent the excretion of all newly synthesized biomolecules after activation. An aliquot of blood was incubated in a DurActive3 for two hours at 37 °C, and then, to permeabilize the membrane, a fixative solution was added. The entire solution was thus transferred into the Duraclone IF Monocyte Activation Tube (Beckman Coulter Inc., Brea, CA, USA) in which the specific monoclonal antibodies bound to fluorochromes (HLA-DR-RPE/TNFα-AF700/CD14-PB/CD45-KRO) are present. After 15 min of incubation in the dark, we proceeded with the acquisition using a flow cytometer. Following manufacturer’s instructions, the gating strategy was applied as follows: the discriminator on the FS parameter was set to an as much as low a value as possible to ensure that the leukocytes were not excluded from the acquisition. A CD45-KRO Vs. SSC dot plot was created and, within it, a gate was created to enclose the CD45+. An additional CD14-PB Vs. SSC dot plot was set and, on this, a gate was created to enclose the CD14+ CD45+ monocytes. The expression of iTNF-A+ and HLA-DR+ was evaluated on the CD45+ CD14+ population. All results were expressed as the percentages of the TNF-positive monocytes (% positive cells). The HLA-DR expression on the monocytes was then measured on their surface (mono-parametric histogram) as the median of the fluorescence intensity and were shown as the % of positive cells. All samples were analyzed no later than 5 h after the execution of the venous sampling.

### 2.3. Statistical Analysis

Data obtained from the analysis were analyzed for statistical significance using the GraphPad 9.0.0 software. Patient baseline characteristics were described using descriptive statistics. Pearson’s test was used to evaluate the distribution and a value of *p*-value < 0.05 confirmed a Gaussian distribution of the data. The difference between the groups was evaluated graphically using box and whiskers plots. The comparison between the groups is reported as mean ± SD. Statistical significance was obtained through paired or unpaired Student’s *t*-tests. In all the of the analyses, *p*-values < 0.05 were considered as statistically significant. The best cut-off was derived using the Cutoff Finder software, a freely available web application enabling rapid biomarker cutoff optimization (http://molpath.charite.de/cutoff, accessed on 8 October 2020).

## 3. Results

### 3.1. Patient Characteristics

Patient demographics are summarized in Table 1. For this study, a total of twenty-five patients were considered. The median age of all of the patients was 61 (range 34–78). Consistent with emerging evidence [36,37] that men are more likely to develop serious disease, 80% (N = 20) of the patients in the cohort were male, and 20% (N = 5) were female. The median time for subjects requiring oxygen therapy or mechanical ventilation, either non-invasive or invasive (intubation), was 10 days (range 5–17). The patients were classified on the basis of their outcome: 9 out of 25 (36%) died (Exitus Group), and 16 (64%) recovered from COVID-19 (Good Prognosis Group). The exitus group was composed of patients who never recovered from COVID-19 and who died between day 10 and day 20 after hospitalization. The Good Prognosis Group was composed of patients who recovered from COVID-19 with a test that was negative for COVID-19 infection at the time of discharge from the hospital, usually between day 15 and day 20 of hospitalization. As COVID-19 and the related health emergency found that the majority of the hospitals and clinicians around the world were largely unprepared, the management and treatment of patients was incredibly difficult considering the little information on COVID-19 that was available during first emergency situation. In particular, during March and April 2020, no universally approved treatments were available in clinical practice, and the Italian guidelines were constantly being updated. With the exception of oxygen therapy, all of the infected subjects were treated with antiviral drugs (hydroxychloroquine and lopinavir and ritonavir) and antibiotics (azithromycin), and no impact on outcome of the treatments was observed between the two groups.

### 3.2. Hematological and Inflammatory Values

**Figure 1:**
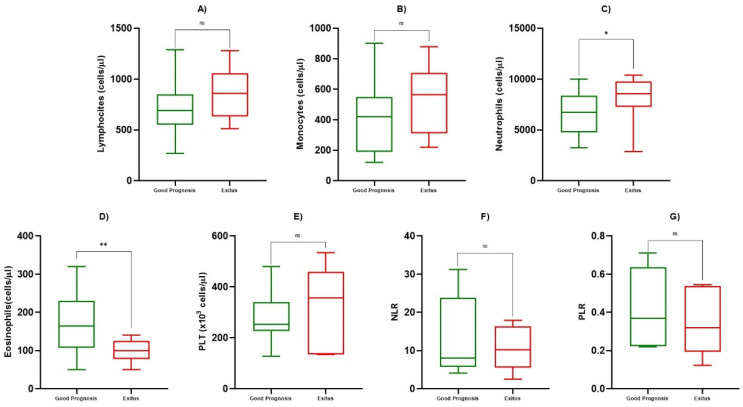
Box and whiskers of blood value. Box and whiskers among good prognosis and exitus patients at first determination for ALC in (**A**), for AMC in (**B**), for ANC in (**C**), for AEC in (**D**), for PLT in (**E**), for NLR in (**F**), and for PLR in (**G**). The significant *p*-values are marked with * (from 0.01 to 0.05) or ** (from 0.001 to 0.01), not significant are marked with ns (≥0.05).

**Figure 2:**
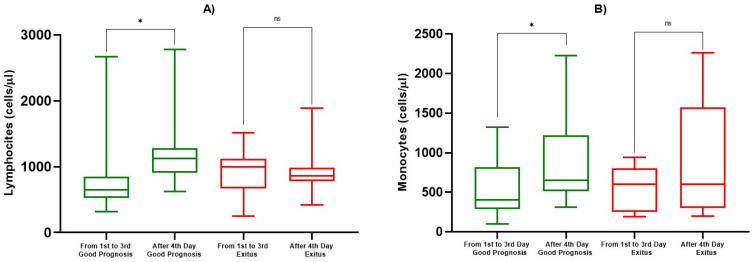
Box and whiskers for ALC and AMC. In (**A**), box and whiskers for ALC and in (**B**), for AMC among good prognosis and exitus patients for determinations between the first and third day and from the fourth day onward. The significant *p*-values are marked with * (from 0.01 to 0.05), not significant are marked with ns (≥0.05).

At admission, we evaluated ALC, AMC, ANC, AEC, PLT, NLR, PLR and, in addition, we analyzed ALC and AMC during the course of infection, for a total of eight determinations. We observed a statistically significant reduction in ALC compared to healthy subjects (817/µL ± 236 vs. 2119/µL ± 1991, *p* < 0.05), but no differences were observed between the Good Prognosis vs. Exitus groups at the first determination (day 0) (*p* = 0.684) (Figure 1a, Table 2). On the other hand, after the fourth determination, we observed a statistically significant increase of ALC towards the normal range in patients with a good prognosis (814/µL ± 546 vs. 1184/µL ± 449, *p* < 0.05), while in Exitus Group, the average remained lower, and no statistically significant differences were observed (925/µL ± 268 vs. 881/µL ± 250, *p* = 0.725) (Figure 2a, Table 3). We also evaluated the AMC at the first day of hospitalization, within the first and third of hospitalization, and beyond the 4th day. At the first determination, patients with a good prognosis and the Exitus Group showed a share of monocytes that were substantially overlapping (*p* = 0.469) (Figure 1b, Table 2), but from the fourth determination onwards, we observed a significant increase in the monocyte count in the Good Prognosis Group (847/µL ± 471 vs. 551/µL ± 367, *p* < 0.05) but not in Exitus Group (*p* = 0.290) (Figure 2b, Table 3). For ANC, AEC, PLT, NLR, and PLR, we only evaluated the value at the first observation. We detected a statistically significant increase of ANC in the Exitus Group compared to the Good Prognosis Group, in which the average was lower (8422/µL ± 3163 vs. 7149/µL ± 2499, *p* < 0.05) (Figure 1c, Table 2). We found a higher statistically significant AEC in the Good Prognosis Group compared to the Exitus Group (179/µL ± 82 vs. 108/µL ± 25, *p* < 0.05) (Figure 1d, Table 2). No differences were observed for PLT (Figure 1e, Table 2). Using the best cut-off value, even if we obtained the best sensibility for PLR (cut-off >8.02, AUC 0.643, sensitivity 100%, specificity 57.1%) and NLR (cut-off ≤0.545, AUC 0.629, sensitivity 100%, specificity 42.9%), no differences were observed for NLR (Figure 1f, Table 2) and PLR (Figure 1g, Table 2) between the Good Prognosis Group vs. the Exitus Group (*p* > 0.05).

### 3.3. TNF-α and Lymphocyte Subpopulations Analysis

**Figure 3:**
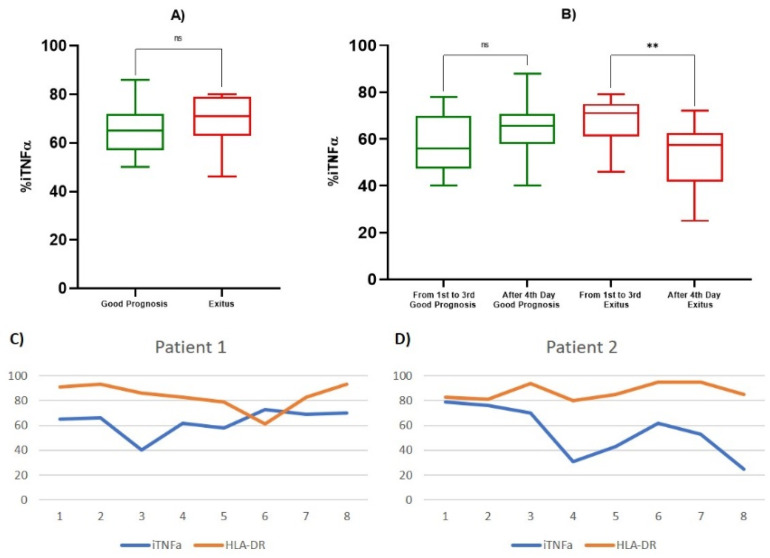
Box and whiskers for iTNF-α and levels of iTNF-α and HLA-DR. (**A**) Box and whiskers for percentage of iTNF-α positive monocytes at first determination in the Good Prognosis and Exitus groups; (**B**) box and whiskers for percentage of iTNF-α positive monocytes in good prognosis and exitus groups for determinations between the first and third day and from the fourth day onward; (**C**) example of iTNF-α and HLA-DR percentage trends in a patient with a good prognosis (total of eight determinations); (**D**) example of the iTNF-α and HLA-DR+ percentage trends in a patient with exitus (total of eight determinations). The significant *p*-values are marked with ** (from 0.001 to 0.01), not significant are marked with ns (≥0.05).

**Figure 4:**
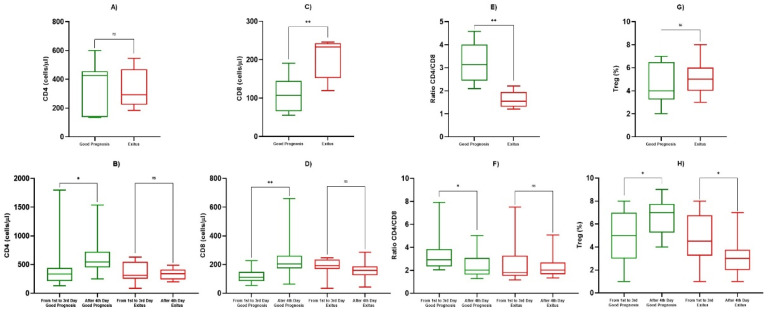
Box and whiskers for lymphocyte subpopulations in the Good Prognosis Group and the Exitus Group. In (**A**), CD4+ T-lymphocytes at the first observation; in (**B**), CD4+ T-lymphocytes for the determinations made between the first and third day and from the fourth day onwards; in (**C**), CD8+ T-lymphocytes at the first observation; in (**D**), CD8+ T-lymphocytes for the determinations made between the first and third day and from the fourth day onwards; in (**E**), CD4+/CD8+ lymphocytes ratio at the first observation; in (**F**), CD4+/CD8+ lymphocytes ratio for the determinations made between the first and third day and from fourth day onwards; in (**G**), % of Tregs at the first observation; in (**H**), % of Tregs for determinations made between the first and third day and from the fourth day onwards. The significant *p*-values are marked with * (from 0.01 to 0.05) or ** (from 0.001 to 0.01), not significant are marked with ns (≥0.05).

All data relating to monocyte iTNF-α and HLA-DR expression on and lymphocyte subpopulations frequency were evaluated using peripheral blood collected from patients on alternate days, starting from hospitalization, for a total of eight determinations. At the first determination, the good prognosis patients showed percentages of iTNF-α positive monocytes (% positive cells) that were lower than those of the Exitus Group, but no statistically significant difference was observed (65.429/µL ± 11.194 vs. 69.200/µL ± 13,570, *p* = 0.469) (Figure 3a, Table 4). Furthermore, we observed that in both groups (Figure 3c,d) in the third or fourth determination, there was a reduction in the percentage of iTNF-α positive monocytes. Subsequently, all patients showed an increased percentage of iTNF-α positive monocytes, which remained prolonged and almost constant in the subjects with a good prognosis (Figure 3c) compared to the Exitus Group (Figure 3d), in which there is a statistically significant decrease in the levels of the iTNF-α positive monocytes between the first and third determinations vs. the fourth to last (67.467/µL ± 10.2878 vs. 53.357/µL ± 14.1073, *p* < 0.05) (Figure 3b, Table 4). In addition, considering the potential role of monocyte HLA-DR in monitoring septic patients [34], we also included the analysis of monocytes that are HLA-DR positive and expressing iTNF-α. The median MFI value of HLA-DR between the two groups was 12.5 for the Good Prognosis Group vs. 12.3 for the Exitus Group. Looking to the % of HLA-DR+ monocytes, no statistically significant difference was observed between the two study groups at the first determination (*p* = 0.684) and between the first and third determinations vs. the fourth to last (*p* = 0.537) (data not shown). Therefore, we evaluated the absolute count of the CD4+ and CD8+ lymphocytes and CD4+/CD8+ lymphocyte ratio in the two groups. At the first determination, the Exitus Group showed a significantly higher count of CD8+ lymphocytes compared to the Good Prognosis Group (204/µL ± 54 vs. 109/µL ± 47, *p* < 0.05) (Figure 4c, Table 4), and no difference was observed for the CD4+ lymphocyte count (*p* = 0.758) (Figure 4a, Table 4). After the fourth determination, in the Good Prognosis Group, we detected a statistically significant increase in the CD4+ (561/µL ± 288 vs. 431/µL ± 383, *p* < 0.05) (Figure 4b, Table 4) and CD8+ (228/µL ± 126 vs. 121/µL ± 51, *p* < 0.05) (Figure 4d, Table 4) counts compared to the values between the first and third determinations (*p* < 0.05), while in the Exitus Group, we did not observe statistically significant differences in the CD4+ and CD8+ counts. Moreover, at the first determination, the Good Prognosis Group showed a higher statistically significant CD4+/CD8+ ratio than the Exitus Group (3.25/µL ± 0.85 vs. 1.61/µL ± 0.37, *p* < 0,05) (Figure 4e, Table 4). Between the first and the third determination vs. after the fourth day, good prognosis patients showed a CD4+/CD8+ ratio that was significantly higher (3.26/µL ± 1.39 vs. 2.87/µL ± 0.63, *p* < 0,05), and no differences were observed for the Exitus Group (*p* = 0.349) (Figure 4f, Table 4). After the evaluation of the basic immunophenotype, we evaluated the Treg compartment. No statistically significant differences were observed in the Treg frequencies at the first determination between the two groups (Figure 4g, Table 4). However, we observed a statistically significant increase in Treg lymphocytes from the fourth determination onwards (4.92/µL ± 2.35 vs. 6.79/µL ± 1.55, *p* < 0.05) in the Good Prognosis Group, while in the Exitus Group, we observed a significant reduction in Treg lymphocytes (4.75/µL ± 2.22 vs. 3.75/µL ± 1.65, *p* < 0.05) (Figure 4h, Table 4). Finally, we evaluated the B and NK lymphocytes subset. No differences were observed for the B lymphocytes between the Good Prognosis and Exitus groups at the first observation (156/µL ± 103 vs. 172/µL ± 117, *p* = 0.651) and among the first and third and after the fourth determination onwards (210/µL ± 127 vs. 196/µL ± 130, *p* = 0.536). We also obtained same results for the NK lymphocytes subset, in which no differences were observed between the two groups at the first observation (184/µL ± 89 vs. 191/µL ± 109, *p* = 0.801) and among the first and third and after the fourth determination onwards (183/µL ± 111 vs. 197/µL ± 139, *p* = 0.793) (data not shown).

## 4. Discussion

The interaction between COVID-19 and the immune system is complex. Infection correlates to an extensive infiltration of the neutrophils and macrophages into the lungs that leads to pro-inflammatory cytokine production, and, in addition, involves CD8+ and CD4+ T cells that orchestrate the immune response against viruses [38]. Unfortunately, hyperactivated neutrophils and monocytes-macrophages are the usual source of the cytokine storm, and a recent study conducted on about 500 COVID-19 infected patients highlighted that the cytokine storm was mediated by high-levels of proinflammatory cytokines and identified that severe cases showed significantly higher cytokines and chemokines such as TNF-α, IL-6, and IL-10 expressed [39]. Monocytes and macrophages may be infected by SARS-CoV-2 through the ACE2-dependent and ACE2-independent pathways. SARS-CoV-2 can effectively suppress the anti-viral IFN response in the monocytes and macrophages. Monocytes in humans are divided into the two main subgroups with different characterization: classical or CD14++CD16- monocytes, known as the most conventional monocytes, and the CD16+ monocyte population that in and of itself consists of intermediate (CD14++CD16+) and non-classical (CD14+CD16++) monocytes [40,41]. It has been discovered that in COVID-19 patients, blood monocytes show a pattern of altered chemokine and cytokine profiles, contributing to a series of inefficient responses, which subsequently enhance the pathogenesis of COVID-19 and leads to an increase in mortality rates [42]. Upon infection, monocytes migrate to the tissues, where they become infected resident macrophages, allowing viruses to spread through all of the organs and tissues. In fact, hyperactivated neutrophils and monocytes-macrophages are the usual source of the cytokine storm and is mediated by high levels of proinflammatory cytokines with significantly higher expression levels of TNF-α, IL-6, and IL-10 [38]. Unfortunately, this potent inflammatory response was not translated into an efficient immune response, but in COVID-19, it can lead to invasive mechanical ventilation and eventually, to the patient’s death. COVID-19 leads to variation in hematological parameters, including the lymphocytes, the white blood cells, the platelets, the neutrophils, etc. [34]. These variations differ on a case to case basis and are also influenced by the level of disease severity. Lymphopenia has been previously reported in about 35–85% of patients and was the most common blood count abnormality. [43] In addition, as previously described for SARS-CoV infected patients [44], a decrease of CD4+ T and CD8+ T cells was also found in the context of COVID-19, and lymphopenia has been implicated as a risk factor for ARDS and mortality, suggesting that the adaptive immune system in the severe infection subgroup was less activated [45]. Furthermore, these patients had lower numbers of Tregs, the absence of which can lead to the production of a cytokine storm and the enhancement of tissue pathology [38]. Overall, those data showed that the dysregulation of the T cells might play a pivotal role in COVID-19 pathogenesis and severity [39]. Considering that the severity of the infection might be described based on differences in lymphocyte subpopulation frequency and laboratory indicators [46], in this retrospective study, we analyzed data relating to 25 COVID-19 infected patients exploring differences in the T, B, NK, and Treg lymphocyte frequencies, differences in ALC, ANC, AMC, AEC, PLT, NLR, and PLR, and differences in the percentages of activated monocytes expressing iTNF-α in order to predict clinical outcome. We found a significant lymphopenia in all COVID-19 infected subjects compared to healthy subjects. In accordance with what has previously been described in the literature [39,45,46], the lymphopenia might be due to the massive activation of lymphocytes in response to the virus. However, as it has emerged from the literature [47,48], in the Good Prognosis Group, we observed an increase in the absolute lymphocyte count, while in the Exitus Group, the lymphopenia remained constant, without showing any improvement during the course of the disease. These findings suggest that lymphopenia might not only be a consequence of the infection but that it is probably a critical factor in driving the development and deterioration of the disease. Therefore, the rise in the absolute lymphocyte count, which in our case occurred around the fourth determination, was a positive prognostic factor. Looking at the neutrophils, at the first determination, we observed that the Exitus Group showed higher neutrophils compared to the patients with a good prognosis. Even if these cells can release a variety of cytokines and chemokines resulting in the regulation of the host immune response, a sustained activation can result in organ damage due to an uncontrolled ROS production and the release of lytic enzymes [49,50]. It has been shown that neutrophils from ARDS patients are hyper-responsive to in vitro stimulation and, with their ability to produce high levels of ROS may play a major role in the pathogenesis of ARDS-associated lung injury and poor patient outcomes [51]. In line with our findings, increasing evidence has highlighted that COVID- 19 patients with a severe infection profile show higher neutrophil counts compared to non-severe groups [52], data that underlined a negative correlation with the outcome. Furthermore, in patients with a good prognosis, we observed a statistically significant increase in the monocyte count during COVID-19 infection. Monocytes and macrophages are highly active secretory cells, contributing to inflammation, innate and adaptive cellular and humoral immunity, and host defenses. While promoting host defenses, tissue homeostasis, and repair, they might also induce tissue damage. It has been shown that monocytes/macrophages removing debris and necrotic tissue through phagocytosis are central to the cellular processes contributing to recovery from COVID-19 infection [53,54,55]. In this scenario, considering that blood monocytes provide a window for macrophage distribution and activation, we evaluated circulating monocytes, and our results indicate a positive correlation between an increased number of monocytes during infection and a favorable outcome. When evaluating AEC the at first determination, we observed higher AEC values in the Good Prognosis Group compared to the Exitus Group. These effector cells can synthesize, store, and release a variety of cytokines, chemokines, and growth factors and can act as antigen presenting cells to regulate the immune capabilities of T lymphocytes [56]. Studies conducted over the past 30 years and more recently on COVID-19 infected patients underlined a vital role of the eosinophils in the host’s defense [33,57,58]. In particular, COVID-19 infected patients showed lower AEC values compared to healthy subjects and that AEC gradually underwent a dynamic recovery process to normal as the patient’s condition improved, while in severe or deceased patients, it remained decreased or continued to dramatically decrease [33,58]. Additionally, in our setting, we found a positive correlation between higher eosinophils and a favorable outcome. No differences were observed for the B and NK lymphocytes or for NLR, PLT, and PLR between the two groups. Regarding T lymphocytes, during the first determination, we observed higher levels of CD8+ T cells in the Exitus Group and no difference in the CD4+ T lymphocytes. During the course of infection, an increase of CD4+ and CD8+ T lymphocytes counts during the fourth determination in the Good Prognosis Group, while in the Exitus Group, even if not statistically significant, we observed a decrease for both the CD4 and CD8 counts. At the same time, patients with a good prognosis showed a CD4+/CD8+ ratio that was significantly higher than the Exitus Group during the first determination and, among the first and the third vs. After the fourth determinations, the good prognosis patients showed a CD4+/CD8+ ratio that was significantly higher. T lymphocytes play a crucial role in viral clearance, and in particular, CD8+ cytotoxic T lymphocytes secrete key molecules such as perforin, granzyme, and IFN-γ for this purpose [55]. At the same time, the CD4+ helper T cells support cytotoxic T lymphocytes and B lymphocytes by enhancing their ability to eliminate pathogens [59,60]. However, massive stimulation by the virus can induce cellular exhaustion, resulting in the loss of cytokine production capacity and functional reduction [15]. A decrease of CD4+ T and CD8+ T cells was found in the context of COVID-19, and lymphopenia has been implicated as a risk factor for ARDS and mortality, suggesting that the adaptive immune system in the severe infection subgroup was less activated [44,61,62]. It has been reported that the counts rise dramatically in patients with attenuated symptoms or improved radiological abnormalities [63,64]. However, both the CD4+ T and CD8+ T counts may serve as biomarkers for predicting the severity and recovery from COVID-19 [65], and our results underlined a negative correlation with the outcome of the CD8+ T lymphocyte count at the first observation, but on the other hand, they also highlighted a positive correlation between a rise in CD4+ and CD8+ during infection and in the CD4+/CD8+ T lymphocyte ratio and a favorable outcome. Concerning the Treg compartment, we observed a significant increase in Treg lymphocytes during the course of the disease in Good Prognosis Group, while within the Exitus Group, we observed a significant reduction. In COVID-19 infected patients, it has been shown that the number of circulating Treg cells is reduced in patients compared to in control groups and in severe COVID-19 patients, suggesting the important role of Treg cells in disease progression and immune system regulation [66,67]. Aligned with the literature, our characterization of the Tregs showed a positive relationship between the rise in absolute the Treg count during the course of disease and outcome. 

Alteration in monocyte count, phenotype, and function plays a significant role in the SARS-CoV-2 pathogenesis, as reported in many studies, and depends on the stage of the disease and is variable in different manuscripts [68,69]. In COVID-19 patients, the cytokine storm was mediated by high-levels of proinflammatory cytokines, and the severe cases showed significantly higher cytokines and chemokines, such as TNF-α, IL-6, and IL-10 expressed [39,59]. We evaluated the ability of the activated monocytes to express TNF-α cytokine together with the monocyte absolute count. Our analysis highlighted no statistically significant differences in the percentages of iTNF-α positive activated monocytes between the two groups at the first determination, but a reduction in iTNF-α positive activated monocytes was observed in both groups between the third and fourth determination. After the fourth determination, there was an increase in iTNF-α synthesis in the Good Prognosis Group, which remains substantially constant in subsequent determinations. In the Exitus Group, after the fourth determination, a statistically significant reduction in iTNF-α positive activated monocytes was found. TNF-α is a pro-inflammatory cytokine, and it is responsible for the inflammatory insult that the body undergoes in response to infection, and therefore, therapies that inhibit TNF-α have been hypothesized. However, clinical studies have reported that anti-TNF-α antibody treatments have not had beneficial effects for patients with sepsis [70]. In fact, even if the activation of TNF-α of endothelial cells and leukocytes and the stimulation of signaling pathways downstream can contribute to the inflammatory lesion, these actions are also fundamental for the defense of the host. In line with this role of TNF-α, the rise in activated monocytes expressing iTNF-α during COVID-19 infection probably reflects the increased capacity of monocytes to release proinflammatory cytokines, so their ability to contrast the infections and the related immunosuppression may represent a positive prognostic factor.

Overall, the first COVID-19 wave and the related health emergency have found the majority of the hospitals and clinicians to be unprepared around the world. For this reason, the management of patients and the planning of research analyses on an optimal cohort of infected subject was incredibly difficult considering the little information on COVID-19 that was available during first emergency. It needs to be considered that the 25 patients enrolled in this study were hospitalized during the first COVID-19 wave/emergency, Italy was suggesting that subjects with COVID-19 symptoms remain at home for as long as possible so as to avoid congesting the hospitals due to the lack of hospital beds dedicated to COVID-19 patients. For this reason, all of the patients enrolled in this study were hospitalized as a consequence of their respiratory insufficiency and without a previous COVID-19 test. Once they were admitted to the hospital, they were tested for COVID-19 infection, and the test results showed that the participants were positive for COVID-19 infection. As a result, the day of onset corresponds to day 0/the first determination, and for this reason, we did not evaluate the impact of the time from symptom onset to admission to the hospital based on the severity of the illness. Considering these points of view, our preliminary results need to be validated in a larger, homogeneous and well-defined cohort of patients. Additionally, the potential drawbacks of our study remain due to the fact that we only included 25 patients, and we did not include a cohort of healthy patients on which we could have conducted the same analyses. We also did not provide a comparison with soluble TNF-α or others soluble mediators in whole blood.

## 5. Conclusions

Many studies have revealed that monocytes and macrophages can induce pathogenesis during the COVID-19 through dysregulated function, including the enhancement of inflammatory cytokines and chemokines. For these reasons, discovering the involved mechanisms and mediators can be significantly helpful to develop novel therapeutic methods and cell-based drugs for alleviating SARS-CoV-2 infection. Considering that COVID-19 disease has a well-determined phasic progression and rapidly deteriorates approximately seven days after the onset of symptoms, it is crucial to identify predictive clinical biomarkers as early as possible to better assist patients. In this vision, we have done a preliminary longitudinal analysis on COVID-19 infected patients, evaluating the rise and fall of different mediators of the immune system during infection. The preliminary data collected in our study point to a modulation of the different cellular and soluble mediators of the immune system during infection, which probably play a key role in the outcome of COVID-19. Based on our preliminary findings, further studies are needed to better understand the roles and balance of the different immune cell subsets involved in the protection or pathogenesis of COVID-19 in order to detect the clinical signs that are predictive of outcome as early as possible.

## Figures and Tables

**Table 1:** Patient demographics. For this study, a total of twenty-five patients diagnosed with SARS-COV-2 infection were enrolled and classified into two group on the basis of their outcome: the Exitus Group (patients who died while still positive to Covid-19) and the Good Prognosis Group (patients recovered from Covid-19 and who are still alive).

Study Population	ALL Patients N. 25 (100%)	Good Prognosis N. 16 (76%)	Exitus N. 9 (24%)
Female/Male, n (%)	5 (20)/20 (80)	3 (19)/13 (81)/	2 (22)/7 (78)
Median Age (Range)	61 (34–78)	64 (34–71)	59 (48–67)
Median Days Oxygen Therapy (Range)	10 (5–17)	7 (5–12)	13 (8–17)
Median P/F (Range)	165 (60–338)	139 (60–276)	200 (80–338)
Subjects Without Comorbidity, n (%)	14 (56)	11 (78)	3 (22)
Subjects With ONE Comorbidity, n (%)	7 (28)	3 (42)	4 (58)
Subjects With TWO Comorbidities, n (%)	4 (16)	1 (25)	3 (75)
Diabetes, n	2	0	2
Hypertension, n	10	4	6
Chronic Liver Disease, n	1	0	1
Chronic Pulmonary Disease, n	1	0	1
Chronic Renal Disease, n	1	1	0

**Table 2:** Analysis of ALC, AMC, ANC, AMC, AEC, PLT, NLR, and PLR. Mean, deviation standard, and *p*-value at first observation among the Good Prognosis Group and the Exitus Group. The significant *p*-values are shown in bold.

Parameter at First Observation	Good Prognosis Mean ± SD	Exitus Mean ± SD	*p*-Value
ALC (cells/µL)	767 ± 262	846 ± 241	0.684
AMC (cells/µL)	438 ± 255	540 ± 208	0.469
ANC (cells/µL)	7149 ± 2499	8422 ± 3163	**0.0161**
AEC (cells/µL)	179 ± 82	108 ± 25	**0.0033**
PLT (cells/µL)	276 ± 109	308 ± 172	0.569
NLR	13.16 ± 10.33	10.77 ± 5.89	0.935
PLR	0.440 ± 0.206	0.357 ± 0.180	0.464

**Table 3:** Analysis of ALC and AMC at different time points (days of hospitalization). Mean, deviation standard, and p-value at first observation, between the first and third observation, and from the fourth observation onwards among the Good Prognosis Group and the Exitus Group. The significant *p*-values are shown in bold.

Parameter	Determination Points	Good Prognosis Mean ± SD	*p*-Value	Exitus Mean ± SD	*p*-Value
ALC (cells/µL)	1st–3rd Day	814 ± 546	**0.0193**	925 ± 268	0.725
	After 4th Day	1184 ± 449		881 ± 250	
AMC (cells/µL)	1st–3rd Day	551 ± 367	**0.0339**	550 ± 282	0.290
	After 4th Day	847 ± 471		908 ± 699	

**Table 4:** Analysis of iTNF-α, CD4+ lymphocytes, CD8+ lymphocytes, CD4+/CD8+ lymphocytes ratio, and Tregs at different time points. Mean, deviation standard, and *p*-value at first observation, between the first and third observation, and from the fourth observation onwards among good prognosis and exitus patients. The significant *p*-values are shown in bold.

Parameter	Determination Points	Mean ± SD	*p*-Value
iTNF-α %	1st Observation (Good Prognosis)	65.429 ± 11.914	0.469
	1st Observation (Exitus)	69.200 ± 13.570	
	1st–3rd Day (Good Prognosis)	58.111 ± 12.6207	0.432
	After 4th Day (Good Prognosis)	64.167 ± 10.9450	
	1st–3rd Day (Exitus)	67.467 ± 10.2878	**0.0046**
	After 4th Day (Exitus)	53.357 ± 14.1073	
CD4+ (cells/µL)	1st Observation (Good Prognosis)	367 ± 426	0.758
	1st Observation (Exitus)	334 ± 291	
	1st–3rd Day (Good Prognosis)	431 ± 383	**0.045**
	After 4th Day (Good Prognosis)	561 ± 288	
	1st–3rd Day (Exitus)	363 ± 162	0.748
	After 4th Day (Exitus)	334 ± 91	
CD8+ (cells/µL)	1st Observation (Good Prognosis)	109 ± 47	**0.0085**
	1st Observation (Exitus)	204 ± 54	
	1st–3rd Day (Good Prognosis)	121 ± 51	**0.0017**
	After 4th day (Good Prognosis)	228 ± 126	
	1st–3rd Day (Exitus)	181 ± 67	0.151
	After 4th Day (Exitus)	160 ± 57	
Ratio CD4+/CD8+	1st Observation (Good Prognosis)	3.25 ± 0.85	**0.0026**
	1st Observation (Exitus)	1.61 ± 0.37	
	1st–3rd Day (Good Prognosis)	3.26 ± 1.39	**0.0209**
	After 4th Day (Good Prognosis)	2.87 ± 0.63	
	1st–3rd day (Exitus)	2.16 ± 0.95	0.349
	After 4th Day (Exitus)	2.28 ± 0.88	
Tregs (cells/µL)	1st Observation (Good Prognosis)	4.83 ± 1.89	0.884
	1st Observation (Exitus)	4.92 ± 1.58	
	1st–3rd Day (Good Prognosis)	4.92 ± 2.35	**0.0429**
	After 4th Day (Good Prognosis)	6.79 ± 1.55	
	1st–3rd Day (Exitus)	4.75 ± 2.22	**0.0426**
	After 4th Day (Exitus)	3.75 ± 1.65	

## Data Availability

The datasets used and/or analyzed during the current study are available from the corresponding author upon reasonable request.
